# Correlation between the genetic variants of base excision repair (BER) pathway genes and neuroblastoma susceptibility in eastern Chinese children

**DOI:** 10.1002/cac2.12088

**Published:** 2020-08-11

**Authors:** Zhenjian Zhuo, Chunlei Zhou, Yuan Fang, Jinhong Zhu, Hongting Lu, Haixia Zhou, Haiyan Wu, Yizhen Wang, Jing He

**Affiliations:** ^1^ Department of Pediatric Surgery Guangzhou Institute of Pediatrics Guangdong Provincial Key Laboratory of Research in Structural Birth Defect Disease Guangzhou Women and Children's Medical Center Guangzhou Medical University Guangzhou Guangdong 510623 P. R. China; ^2^ Department of Pathology Children's Hospital of Nanjing Medical University Nanjing Jiangsu 210008 P. R. China; ^3^ Department of Pathology Anhui Provincial Children's Hospital Hefei Anhui 230051 P. R. China; ^4^ Department of Clinical Laboratory Molecular Epidemiology Laboratory Harbin Medical University Cancer Hospital Harbin Heilongjiang 150040 P. R. China; ^5^ Department of Pediatric Surgery the Affiliated Hospital of Qingdao University Qingdao Shandong 266000 P. R. China; ^6^ Department of Hematology the Second Affiliated Hospital and Yuying Children's Hospital of Wenzhou Medical University Wenzhou Zhejiang 325027 P. R. China

**List of Abbreviations**
APEX1: apurinic/apyrimidinic endonuclease
BER: base excision repair
CI: confidence interval
CNS: central nerve system
eQTL: expression quantitative trait loci
FADS1: fatty acid desaturase 1
FADS2: fatty acid desaturase 2
FEN1: flap endonuclease 1
FPRP: false‐positive report probability
GTEx: Genotype‐Tissue Expression
GWAS: genome‐wide association study
HWE: Hardy‐Weinberg equilibrium
LIG3: DNA ligase III
N/A: not applicable
OGG1: 8‐oxoguanine DNA glycosylase 1
OR: odds ratio
PARP1: poly(ADP)ribose polymerase 1
SNP: single nucleotide polymorphism
TMEM258: transmembrane protein 258
XRCC1: x‐ray repair cross‐complementing group 1

Dear Editor,

Neuroblastoma is the most common non‐central nerve system (CNS) solid tumor in pediatrics [1]. Neuroblastoma accounts for approximately 8% of all pediatric cancers but disproportionally causes a high cancer mortality (15%) in children [2]. Pediatric patients with low‐risk neuroblastoma witness a 5‐year overall survival rate > 90%, whereas the 5‐year overall survival rate in high‐risk neuroblastoma pediatric patients is < 40% [3].

Genetic susceptibility to neuroblastoma is a promising area of research and needs to be fully investigated. For sporadic neuroblastoma, genome‐wide association studies (GWASs) have identified over a dozen causal genetic loci. Studies of candidate genes also reported a decent number of variants predisposing to neuroblastoma. However, the known genetic alternations still could not unveil the full genetic underpinnings of neuroblastoma.

The base excision repair (BER) pathway, one of the DNA repair systems, is responsible for repairing numerous oxidized and alkylated bases by recognizing and excising damaged bases [4]. Many core proteins are involved in the BER pathway, including poly(ADP)ribose polymerase 1 (PARP1), human 8‐oxoguanine DNA glycosylase (OGG1), flap endonuclease 1 (FEN1), apurinic/apyrimidinic endonuclease 1 (APEX1), DNA ligase III (LIG3), and x‐ray repair cross‐complementing group 1 (XRCC1). OGG1 is a bifunctional enzyme (DNA glycosylase and AP lyase) that incises at abasic sites via an AP lyase activity, leaving a single‐strand DNA break intermediate. APEX1 initiates the repair of abasic sites in DNA by cleaving the phosphodiester backbone 5′ to an AP site, creating a nick in the DNA backbone. FEN1 participates in the penultimate steps of Okazaki fragment maturation and 5′‐flap removal during long‐patch BER. LIG3 catalyzes the last stage of BER by sealing the gap. XRCC1 and PARP1 serve as the scaffold protein. Intensive evidence suggests that aberrant BER pathway proteins result in a variety of diseases, especially cancers [4]. Single nucleotide polymorphisms (SNPs) of the BER pathway genes are associated with the risk of various cancer types. Functional analysis revealed that SNPs in the BER pathway genes may modify the kinetics of BER proteins and the DNA repair capacity of the BER system, ultimately affecting carcinogenesis [4]. However, evidence regarding the role of BER pathway gene SNPs in the risk of neuroblastoma waits to be added. To identify more neuroblastoma susceptibility variations in the BER pathway genes, we performed a case‐control study in children at three center hospitals in East China.

This study was conducted in Children's Hospital of Nanjing Medical University (Nanjing, Jiangsu), Anhui Provincial Children's Hospital (Hefei, Anhui), and Yuying Children's Hospital of Wenzhou Medical University (Wenzhou, Zhejiang) in East China. A total of 313 neuroblastoma pediatric patients and 762 cancer‐free children were recruited in this study. The characteristics of the study subjects are summarized in Supplementary Table S1. Age (*P *= 0.823) and gender (*P *= 0.610) were distributed equivalently between the two groups. The study design and participant recruitment were described in our previous work [5].

We successfully genotyped 20 SNPs from 6 BER pathway genes in 313 neuroblastoma pediatric patients and 762 control children (Table 1). Specifically, 3 *PARP1*, 3 *OGG1*, 2 *FEN1*, 3 *APEX1*, 3 *LIG3*, and 6 *XRCC1* SNPs were genotyped. The genotypic distributions of all candidate SNPs were in Hardy‐Weinberg equilibrium (*P* ≥ 0.05) in the controls. The rs174538 of the *FEN1* gene was associated with decreased neuroblastoma risk under the dominant model (adjusted odd ratio [OR] = 0.71, 95% confidence interval [CI] = 0.54‐0.93, *P *= 0.012). However, no significant associations with neuroblastoma risk were found for the remaining SNPs in the single‐locus analysis (all *P* ≥ 0.05; Supplementary Figure S1).

**TABLE 1:** Association between the SNPs in BER pathway genes and neuroblastoma susceptibility in eastern Chinese children

		Allele		Neuroblastoma patients				Cancer‐free controls										
Gene	SNP	A	B	AA	AB	BB	Undetectable	AA	AB	BB	Undetectable	Adjusted OR (95% CI)^a^	*P* value^a^	Adjusted OR (95% CI)^b^	*P* value^b^	Adjusted OR (95% CI)^c^	*P* value^c^	HWE
*PARP1*	rs1136410	A	G	102	151	59	1	240	387	135	0	1.03 (0.70‐1.51)	0.893	0.95 (0.72‐1.26)	0.714	1.08 (0.77‐1.52)	0.660	0.328
	rs2666428	T	C	198	97	17	1	461	262	39	0	1.01 (0.56‐1.83)	0.982	0.88 (0.67‐1.15)	0.343	1.06 (0.59‐1.91)	0.840	0.822
	rs8679	A	G	269	40	3	1	651	107	4	0	1.77 (0.39‐7.96)	0.459	0.92 (0.62‐1.35)	0.672	1.79 (0.40‐8.08)	0.447	0.860
*OGG1*																		
	rs1052133	G	C	128	139	45	1	284	368	110	0	0.91 (0.60‐1.36)	0.634	0.85 (0.65‐1.11)	0.241	1.00 (0.69‐1.46)	1.000	0.600
	rs159153	T	C	260	50	2	1	614	142	6	0	0.79 (0.16‐3.94)	0.770	0.84 (0.59‐1.19)	0.330	0.81 (0.16‐4.06)	0.799	0.478
	rs293795	A	G	275	36	1	1	691	69	2	0	1.17 (0.11‐13.01)	0.897	1.31 (0.86‐1.99)	0.216	1.14 (0.10‐12.63)	0.915	0.842
*FEN1*																		
	rs174538	A	G	142	131	40	0	281	362	119	0	0.67 (0.45‐1.02)	0.059	0.71 (0.54‐0.93)	0.012	0.80 (0.54‐1.17)	0.248	0.893
	rs4246215	T	G	126	140	47	0	278	365	119	0	0.89 (0.60‐1.33)	0.568	0.87 (0.66‐1.13)	0.294	0.97 (0.67‐1.40)	0.868	0.964
*APEX1*																		
	rs1130409	T	G	109	143	61	0	271	346	145	0	1.03 (0.71‐1.50)	0.870	1.02 (0.78‐1.35)	0.874	1.02 (0.73‐1.43)	0.904	0.067
	rs1760944	T	G	101	163	49	0	250	372	140	0	0.87 (0.58‐1.29)	0.486	1.02 (0.77‐1.35)	0.899	0.83 (0.58‐1.19)	0.307	0.937
	rs3136817	T	C	250	60	3	0	630	124	8	0	0.98 (0.26‐3.74)	0.977	1.21 (0.86‐1.69)	0.273	0.95 (0.25‐3.60)	0.935	0.496
*LIG3*																		
	rs1052536	C	T	156	136	21	0	381	316	65	0	0.79 (0.46‐1.33)	0.369	1.01 (0.77‐1.31)	0.963	0.77 (0.46‐1.28)	0.310	0.964
	rs3744356	C	T	307	6	0	0	737	25	0	0	N/A	N/A	0.59 (0.24‐1.45)	0.251	N/A	N/A	0.645
	rs4796030	A	C	101	147	65	0	255	371	136	0	1.19 (0.82‐1.73)	0.367	1.05 (0.79‐1.39)	0.750	1.19 (0.86‐1.66)	0.297	0.958
*XRCC1*																		
	rs1799782	G	A	152	136	25	0	347	336	79	0	0.73 (0.45‐1.20)	0.216	0.90 (0.69‐1.17)	0.424	0.76 (0.47‐1.21)	0.249	0.860
	rs25487	C	T	177	115	21	0	420	302	40	0	1.21 (0.69‐2.11)	0.510	0.93 (0.71‐1.21)	0.574	1.27 (0.73‐2.19)	0.397	0.129
	rs25489	C	T	250	60	3	0	603	153	6	0	1.24 (0.31‐5.00)	0.765	0.94 (0.68‐1.31)	0.723	1.26 (0.31‐5.06)	0.750	0.271
	rs2682585	G	A	245	61	7	0	600	155	7	0	2.51 (0.87‐7.25)	0.089	1.03 (0.75‐1.42)	0.869	2.53 (0.88‐7.30)	0.085	0.384
	rs3810378	G	C	168	122	23	0	398	320	44	0	1.22 (0.71‐2.08)	0.476	0.94 (0.72‐1.22)	0.618	1.28 (0.76‐2.15)	0.363	0.050
	rs915927	T	C	239	73	1	0	613	142	7	0	0.37 (0.05‐3.06)	0.360	1.28 (0.93‐1.76)	0.128	0.35 (0.04‐2.89)	0.332	0.698

a Adjusted for age and gender for homozygous model (BB vs. AA).

b Adjusted for age and gender for dominant model (BB/AB vs. AA).

c Adjusted for age and gender for recessive model (AA/AB vs. BB).

Abbreviations: BER, base excision repair; SNP, single nucleotide polymorphism; OR, odds ratio; CI, confidence interval; HWE, Hardy‐Weinberg equilibrium; N/A, not applicable; *PARP1*, poly(ADP)ribose polymerase 1; *OGG1*, human 8‐oxoguanine DNA glycosylase; *FEN1*, flap endonuclease 1; *APEX1*, apurinic/apyrimidinic endonuclease; *LIG3*, *DNA ligase III; XRCC1*, x‐ray repair cross‐complementing group 1.

We conducted the stratified analyses (Supplementary Table S2) to eliminate potential influences of *FEN1* genotypes on neuroblastoma susceptibility by adjusting confounding factors (age, gender, and site of tumor origin). The protective role of rs174538 AG/GG in decreasing neuroblastoma risk was found in subgroups of age ≤18 months (adjusted OR = 0.60, 95% CI = 0.40‐0.89, *P *= 0.011), females (adjusted OR = 0.59, 95% CI = 0.40‐0.87, *P *= 0.009), and tumors arising from the mediastinum (adjusted OR = 0.53, 95% CI = 0.35‐0.81, *P *= 0.003). Combined analysis stated that the 2 protective genotypes (rs174538 AG/GG and rs4246215 TG/GG genotypes) also decreased neuroblastoma risk in the following subgroups: age ≤ 18 months (adjusted OR = 0.62, 95% CI = 0.42‐0.93, *P *= 0.019), females (adjusted OR = 0.61, 95% CI = 0.41‐0.91, *P *= 0.015), and tumors originated from the mediastinum (adjusted OR = 0.54, 95% CI = 0.36‐0.83, *P *= 0.005).

We carried out false‐positive report probability (FPRP) analysis to validate significant associations (Supplementary Table S3). The threshold for FPRP was preset as 0.2. At the prior probability level of 0.1, significant associations with *FEN1* rs174538 A > G (GG/AG *vs*. AA) remained noteworthy in all subjects (FPRP = 0.121) as well as in the subgroups of females (FPRP = 0.185) and tumors originating from the mediastinum (FPRP = 0.160). In the combined analysis, significant findings for 2 *vs*. 0‐1 protective genotypes (FPRP = 0.166) and its subgroup tumors originated from the mediastinum (FPRP = 0.183) could be called noteworthy.

We further explored the biological effects of *FEN1* rs174538 A > G on the neighboring gene expression by using released data from Genotype‐Tissue Expression (GTEx) Portal (https://www.gtexportal.org/). We observed that rs174538 A allele was significantly associated with increased mRNA expression levels of fatty acid desaturase 2 (*FADS2*) and transmembrane protein 258 (*TMEM258*) in the whole blood, nerve‐tibial, and cell‐cultured fibroblasts (Figure 1A). The rs174538 A allele was also associated with increased expression of fatty acid desaturase 1 (*FADS1*) mRNA in the whole blood, but with decreased expression of *FADS1* mRNA in the nerve‐tibial (Figure 1B).

**FIGURE 1:**
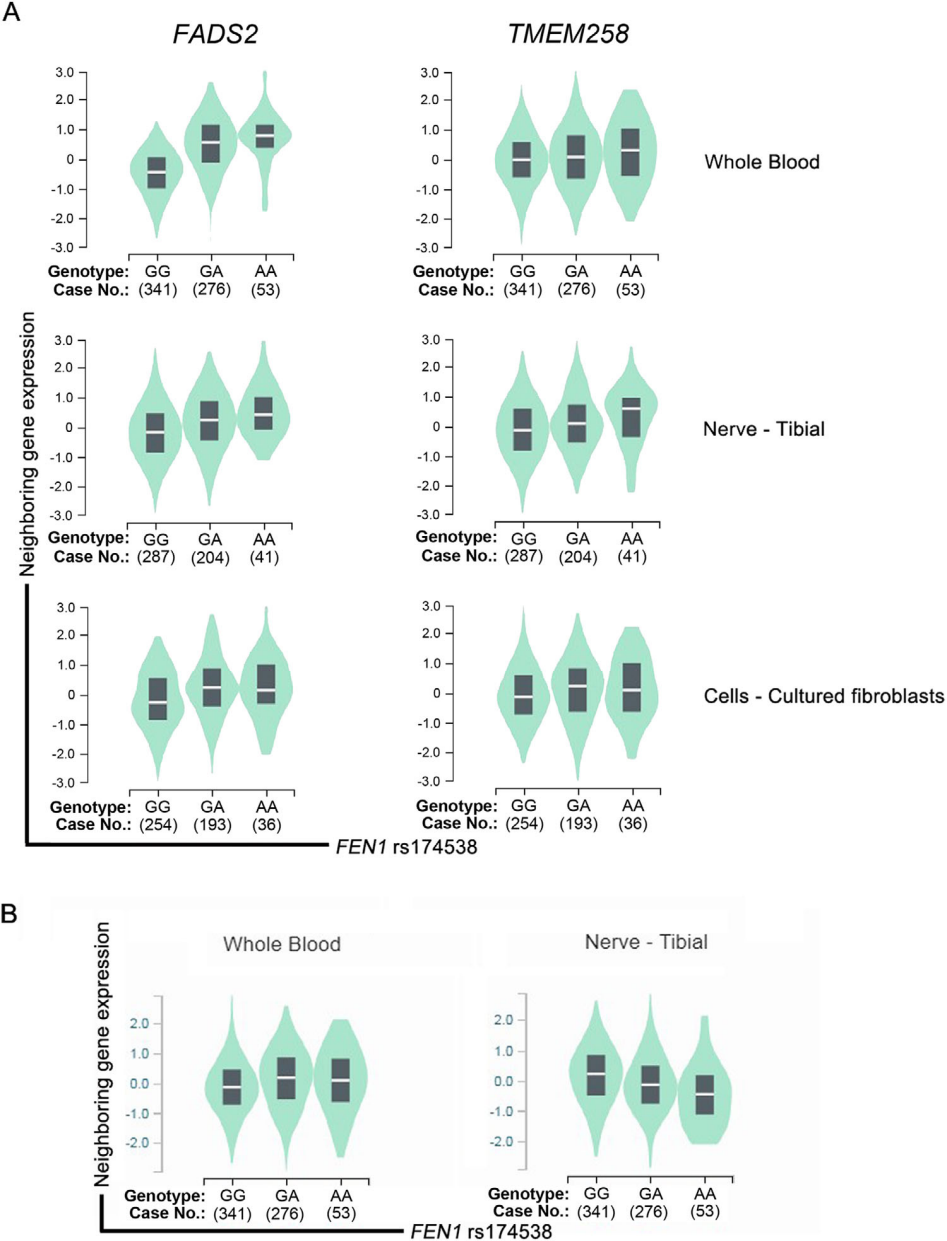
eQTL analysis of the neuroblastoma risk factor *FEN1* rs174538 A > G. **A**. *FADS2* and *TMEM258* levels in the whole blood, nerve‐tibial, and cell‐cultured fibroblasts; **B**. *FADS1* level in the whole blood and nerve‐tibial. Abbreviations: eQTL, expression quantitative loci; *FEN1*, flap endonuclease 1; *FADS2*, fatty acid desaturase 2; *TMEM258*, transmembrane protein 258

The implication of the BER pathway gene SNPs in cancer susceptibility has been highly documented. Plenty of SNPs within the BER pathway genes were found to predispose to various types of cancer. Our group previously carried out a study on BER gene polymorphisms and Wilms tumor susceptibility [6]. Significant associations with Wilms tumor susceptibility were shown for the *OGG1* rs1052133, *FEN1* rs174538, and *FEN1* rs4246215 polymorphisms. Regarding the association of the BER pathway gene SNPs with neuroblastoma risk, only 3 studies were available by far; and all of them were performed by our research group. In these studies, we found that, none of the studied *APEX1* polymorphisms were associated with neuroblastoma risk [5]. Such a negative association was also observed between neuroblastoma risk and polymorphisms in the *OGG1* [7] and *LIG3* genes [8]. However, all these studies were conducted to analyze a single gene in the BER pathway, and the results need to be validated in another independent study. Thus, here we attempted to validate the previous studies by adopting a systematical analysis of potentially functional SNPs in 6 core genes in the BER pathway. In the current study, no significant relationships were detected between neuroblastoma risk and the SNPs in *PARP1*, *OGG1*, *APEX1*, *LIG3*, and *XRCC1* genes. Such results strengthen the previous findings that these variations may be too weak to impact neuroblastoma risk. To be noted, significant conferring roles of the same BER SNPs to the risk of other cancer types have been detected, such as *PARP1* rs1136410 and thyroid cancer [9], *OGG1* rs1052133 and Wilms tumor [6], *FEN1* rs4246215 and Wilms tumor [6], *APEX1* rs1130409 and renal cell carcinoma [10], *LIG3* rs1052536 and lung cancer [11]. The different roles of these SNPs in specific cancer types indicated that specific cancer types should be set before interpreting the role of SNPs.

Excitedly, we demonstrated that the rs174538 of the *FEN1* gene could protect from neuroblastoma. FEN1 is a structure‐specific nuclease involved in the removal of 5′‐flap during long‐patch BER and the maturation of Okazaki fragments in DNA replication. Moreover, FEN1 is also characterized as a 5′ exonuclease and a gap‐dependent endonuclease, which mediates apoptotic DNA degradation during apoptosis. The *FEN1* gene is mapped to chromosome 11 (11q12.2). Yang et al. [12] identified that the rs174538 A allele of the *FEN1* gene decreased risk for lung cancer by decreasing FEN1 expression. Moreover, they detected that coke oven workers who carried the AA genotype have significantly lower DNA damage level than those with GG or GA genotypes. In a meta‐analysis conducted for the overall cancer, the results suggested that the subjects with *FEN1* rs174538 A allele have a decreased susceptibility to cancer in Chinese populations [13]. We further performed online expression quantitative trait loci (eQTL) analysis to interpret the possible mechanism of how rs174538 impacts neuroblastoma risk. eQTL evidence suggested that the A allele in rs174538 was significantly associated with the increased mRNA expression levels of *FADS2* and *TMEM258*. Further functional experiments conducted in neuroblastoma cells are needed to show how the *FEN1* rs174538 A allele can be associated with altered expressions of these genes. *FADS2* was found to function as a potential oncogene in some types of cancer [14]. *TMEM258* is a central mediator of endoplasmic reticulum quality control and intestinal homeostasis, yet its role in cancer remains unknown [15]. The exact relationship of *FADS2* and *TMEM258* with neuroblastoma risk waits to be elucidated. Taken together, the significant role of rs174538 A allele in cancer deserves more attention for further exploration. Although at the preliminary stage, our findings represent a novel mechanism by which rs174538 may modulate the expression of multiple nearby genes, thereby impacting the risk of neuroblastoma.

Our study has several limitations. First, the sample sizes were small in some stratification analyses. Second, the number of analyzed SNPs was limited. Another limitation was the lack of incorporating analysis on environment factors and genetic‐environmental factors. The fourth limitation was that the current study only focused on the subjects of the Han population. Replication of these findings in additional individuals of non‐Chinese descent should be helpful to validate our findings.

In conclusion, we showed a robust association of genetic variants in the *FEN1* gene with neuroblastoma risk in a relatively large sample size of pediatric patients in East China. Intensive future research is warranted to extend the role of *FEN1* gene loci in neuroblastoma susceptibility in individuals of non‐Chinese ancestries.

## DECLARATIONS

### ETHICS APPROVAL AND CONSENT TO PARTICIPATE

The study protocol conformed to the ethical guidelines of the 1975 Declaration of Helsinki, and was approved by the Ethics Committees of Children's Hospital of Nanjing Medical University, Anhui Provincial Children's Hospital, and the Second Affiliated Hospital and Yuying Children's Hospital of Wenzhou Medical University. Each participant signed an informed consent before participating to this study.

### CONSENT FOR PUBLICATION

Not applicable.

### AVAILABILITY OF DATA AND MATERIALS

All data generated or analyzed during this study are included in this published article and its additional files.

### COMPETING INTERESTS

The authors declare that they have no competing interests.

### FUNDINGS

This work was supported by the grants from Natural Science Foundation of Guangdong Province (2019A1515010360) and Guangdong Provincial Key Laboratory of Research in Structural Birth Defect Disease (2019B030301004).

### AUTHORS’ CONTRIBUTIONS

Z.Z., J.Z., H.L., J.H., and Y.W. designed the study, performed the experiments and wrote the manuscript. C.Z., Y.F., H.Z., H.W., and Y.W. collected the clinical samples and information. Z.Z. and J.H. analyzed the data and prepared all the tables and figures. Z.Z., J.H., and Y.W. coordinated the study. All authors reviewed and approved the final manuscript.

## Supporting information

Supporting Information
