# Supplementary material for: Correlation between the genetic variants of base excision repair (BER) pathway genes and neuroblastoma susceptibility in eastern Chinese children
Source: Cancer Commun (Lond). 2020 Aug 11;40(11):641–6. doi: 10.1002/cac2.12088 (PMC7668499; doi:10.1002/cac2.12088)
Supplement: Supplementary file 1 — Supporting Information [file CAC2-40-641-s001.docx]

**Supplementary Table S1.** Demographic characteristics of 313 neuroblastoma patients and 762 cancer-free children from East China.

| **Characteristic** | **Neuroblastoma patients**  **[cases (%)]** | **Cancer-free controls**  **[cases (%)]** | ***P* value ^a^** |
| --- | --- | --- | --- |
| **Age (months)** |  |  | 0.823 |
| ≤18 | 142 (45.37) | 340 (44.62) |  |
| >18 | 171 (54.63) | 422 (55.38) |  |
| **Gender** |  |  | 0.610 |
| Female | 145 (46.33) | 340 (44.62) |  |
| Male | 168 (53.67) | 422 (55.38) |  |
| **Site of tumor origin** |  |  | N/A |
| Adrenal gland | 68 (21.73) | N/A |  |
| Retroperitoneal region | 126 (40.26) | N/A |  |
| Mediastinum | 99 (31.63) | N/A |  |
| Others | 20 (6.39) | N/A |  |

N/A, not applicable.

^a^ Two-sided χ^2^ test between neuroblastoma patients and cancer-free controls.

**Supplementary Table S2**. Stratification analysis for the association between *FEN1* genotypes and neuroblastoma susceptibility in eastern Chinese children.

| **Characteristics** | **rs174538**  **(patient/control)** | | **Adjusted OR**  **(95% CI)^a^** | ***P* value^a^** | **rs4246215**  **(patient/control)** | | **Adjusted OR**  **(95% CI)^a^** | ***P* value^a^** | **Protective genotypes^b^**  **(patient/control)** | | **Adjusted OR**  **(95% CI)^a^** | ***P* value^a^** |
| --- | --- | --- | --- | --- | --- | --- | --- | --- | --- | --- | --- | --- |
|  | **AA** | **AG/GG** |  |  | **TT** | **TG/GG** |  |  | **0-1** | **2** |  |  |
| **Age (months)** | | | | | | | | | | | | |
| ≤18 | 72/129 | 70/211 | **0.60 (0.40-0.89)** | **0.011** | 64/131 | 78/209 | 0.78 (0.52-1.15) | 0.210 | 72/132 | 70/208 | **0.62 (0.42-0.93)** | **0.019** |
| >18 | 70/152 | 101/270 | 0.81 (0.56-1.17) | 0.258 | 62/147 | 109/275 | 0.94 (0.65-1.36) | 0.728 | 70/153 | 101/269 | 0.82 (0.57-1.18) | 0.281 |
| **Gender** | | | | | | | | | | | | |
| Female | 73/125 | 72/215 | **0.59 (0.40-0.87)** | **0.009** | 68/126 | 77/214 | 0.69 (0.46-1.03) | 0.067 | 73/128 | 72/212 | **0.61 (0.41-0.91)** | **0.015** |
| Male | 69/156 | 99/266 | 0.84 (0.58-1.21) | 0.349 | 58/152 | 110/270 | 1.07 (0.73-1.55) | 0.741 | 69/157 | 99/265 | 0.85 (0.59-1.22) | 0.377 |
| **Site of tumor origin** | | | | | | | | | | | | |
| Adrenal gland | 32/281 | 36/481 | 0.66 (0.40-1.09) | 0.102 | 30/278 | 38/484 | 0.73 (0.44-1.21) | 0.224 | 32/285 | 36/477 | 0.68 (0.41-1.11) | 0.123 |
| Retroperitoneal | 50/281 | 76/481 | 0.90 (0.61-1.32) | 0.579 | 46/278 | 80/484 | 1.01 (0.68-1.50) | 0.954 | 50/285 | 76/477 | 0.92 (0.62-1.35) | 0.668 |
| Mediastinum | 52/281 | 47/481 | **0.53 (0.35-0.81)** | **0.003** | 44/278 | 55/484 | 0.72 (0.47-1.11) | 0.135 | 52/285 | 47/477 | **0.54 (0.36-0.83)** | **0.005** |
| Others | 8/281 | 12/481 | 0.90 (0.36-2.23) | 0.817 | 6/278 | 14/484 | 1.39 (0.53-3.67) | 0.507 | 8/285 | 12/477 | 0.92 (0.37-2.29) | 0.862 |

^a^ Adjusted for age and gender, omitting the corresponding stratify factor.

^b^ rs174538 AG/GG and/or rs4246215 TG/GG genotypes.

Abbreviations: OR, odds ratio; CI, confidence interval.

**Supplementary Table S3.** False-positive report probability analysis for the significant findings derived from eastern Chinese children.

| **Characteristics** | **Crude OR (95% CI)** | ***P* value^a^** | **Statistical power^b^** | **Prior probability** | | | | |
| --- | --- | --- | --- | --- | --- | --- | --- | --- |
|  |  |  |  | **0.25** | **0.1** | **0.01** | **0.001** | **0.0001** |
| ***FEN1* rs174538 A>G** | | | | | | | | |
| GG/AG *vs.* AA | 0.70 (0.54-0.92) | 0.010 | 0.643 | 0.044 | 0.121 | 0.601 | 0.938 | 0.993 |
| ≤18 months | 0.59 (0.40-0.88) | 0.010 | 0.279 | 0.096 | 0.242 | 0.778 | 0.973 | 0.997 |
| Female | 0.57 (0.39-0.85) | 0.006 | 0.222 | 0.070 | 0.185 | 0.714 | 0.962 | 0.996 |
| Mediastinum | 0.53 (0.35-0.80) | 0.003 | 0.142 | 0.060 | 0.160 | 0.676 | 0.955 | 0.995 |
| **Protective genotypes (rs174538 AG/GG and rs4246215 TG/GG)** | | | | | | | | |
| 2 protective genotypes *vs.* 0-1 protective genotype | 0.72 (0.55-0.94) | 0.016 | 0.703 | 0.062 | 0.166 | 0.686 | 0.957 | 0.995 |
| ≤18 months old | 0.62 (0.42-0.92) | 0.017 | 0.344 | 0.126 | 0.302 | 0.826 | 0.980 | 0.998 |
| Female | 0.60 (0.40-0.88) | 0.010 | 0.281 | 0.094 | 0.237 | 0.773 | 0.972 | 0.997 |
| Mediastinum | 0.54 (0.36-0.82) | 0.004 | 0.165 | 0.070 | 0.183 | 0.711 | 0.961 | 0.996 |

^a^ Chi-square test was used to calculate the genotype frequency distributions.

^b^ Statistical power was calculated using the number of observations in the subgroup and the OR and *P* values in this table.

Abbreviations: OR, odds ratio; CI, confidence interval.

**
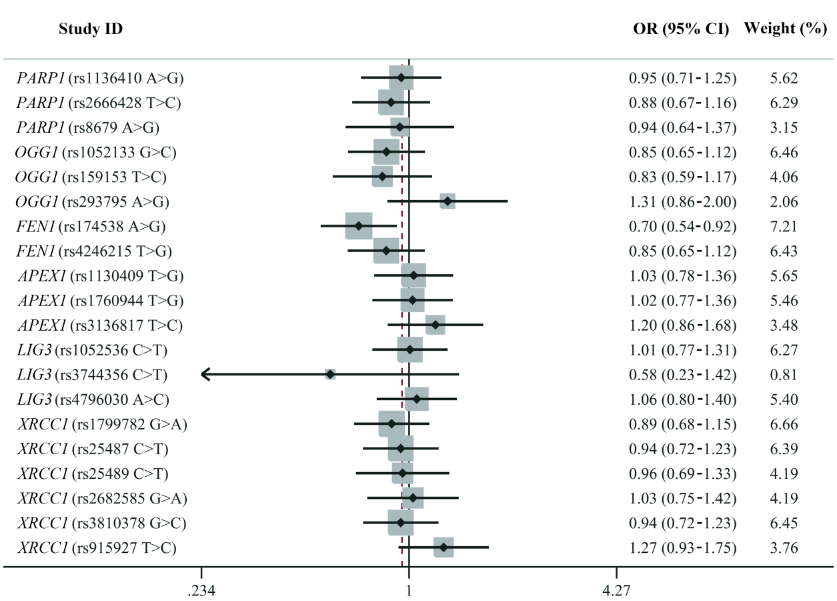
**

**Supplementary Figure S1.** Forest plot for the correlation between the SNPs of BER pathway genes and neuroblastoma susceptibility under the dominant model (BB/AB *vs.* AA). For each SNP, box means the estimate of OR, and horizontal line means 95% CI.

Abbreviations: SNP, single nucleotide polymorphism; BER, base excision repair; OR, odds ratio; CI, confidence interval; *PARP1*, poly(ADP)ribose polymerase 1; *OGG1*, human 8-oxoguanine DNA glycosylase; *FEN1*, flap endonuclease 1; *APEX1*, apurinic/apyrimidinic endonuclease; *LIG3*, DNA ligase III; *XRCC1*, x-ray repair cross-complementing group 1.
